# New System for Measuring Impact Vibration on Floor Decking Sheets

**DOI:** 10.3390/s150100635

**Published:** 2014-12-30

**Authors:** Carlos Moron, Alfonso Garcia, Daniel Ferrandez

**Affiliations:** Sensors and Actuators Group, Department of Tecnología de la Edificación, Universidad Politécnica de Madrid, Madrid 28040, Spain; E-Mails: alfonso.garciag@upm.es (A.G.); daniel.ferrandez.vega@alumnos.upm.es (D.F.)

**Keywords:** vibration, impact, capacitive sensor, elasticity modulus

## Abstract

Currently, there is a narrow range of materials that are used as attenuators of impact noise and building vibrations. Materials used in construction, such as elastic materials, must meet the requirement of having very low elastic modulus values. For the determination of the material's elastic modulus and the acoustic insulation of the same, costly and difficult to execute testing is required. The present paper exposes an alternative system that is simpler and more economic, consisting of a predefined striking device and a sensor able to determine, once the strike is produced, the energy absorbed by the plate. After the impact is produced, the plate undergoes a deformation, which absorbs part of the energy, the remaining part being transmitted to the slab and, at the same time, causing induced airborne noise in the adjoining room. The plate absorbs the power through its own deformation, which is measured with the help of a capacitive sensor. This way, it would be possible to properly define the geometry of the plates, after the execution of the test, and we will try to establish a relationship between the values proposed in this research and the acoustic behavior demanded by the Spanish standards.

## 1. Introduction

In the building field, it is of special interest to know the behavior of different materials against impact noise attenuation. For example, certain elastic coatings or certain constructive solutions, such as floating slabs, enable the reduction of vibration transmissions and provide better soundproofing against this kind of noise [1]. Thus, one of the main parameters of the resistance of materials, especially among construction materials, is the dynamic stiffness, which is obtained from the relation existing between the stress to which a body is subjected and the deformation experienced by the same.

The usual methods performed in the laboratory for the determination of the elastic modulus are based on a static test that measures, with precision, the produced stresses and deformations of the material. There are a large number of studies on the addition of materials to concrete and their reduction of impact noise transmission [2,3]. Furthermore, articles on how to determine the Young's modulus in this material, based on the application of acoustic waves generated from impacts, can be found [4,5], as well as sensors able to determine the position of these impacts on metal parts [6].

However, in the field of prefabricated tiles, studies enabling the determination of the acoustic reduction index, from the striking of a plate with a given load, and depending on the energy absorbed from their deformation, beyond tests with accelerometers proposed by the standard ISO 9052, are few. Dropping a known load from a known height, we can determine the energy introduced to the system. It is easy to measure the capacity of the sensor before the impact and also to determine the capacity of the system during the impact. Thus, with this difference between capacities, obtained as a result of the variation of the gap between the plates of the sensor, the deformation suffered by the plate and the energy that it has absorbed in becoming deformed can be determined.

The main objective of this article is to show the development of a system that allows an alternative test that is faster and more economical than the one proposed by the current standard ISO 10140, which provides reliable results and which can be used as a reference tool before the standardized tests.

## 2. Experimental Process

### 2.1. Determination of the Plate's Shape

One of the main ideas of this paper was to determine, in an analytical way, the most appropriate shape for the plates& footing and, subsequently, to test its suitability for trial execution.

**Figure 1.**
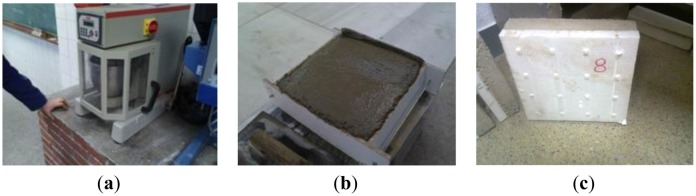
(**a**) Automatic mixer; (**b**) Filled and leveled samples; (**c**) Standard sample tested.

The tiles used are mixed because they are formed by a lower base of EPS (expanded polystyrene) and a top layer of mortar (Figure 1a). Following, the thicknesses and the manufacturing process of each of the components are detailed. Each of them has dimensions of 200 mm × 200 mm (Figure 1b). Since the industrialized production of underfloor heating system is done by a molding process and this requires several months for its implementation, in this paper, it was decided to produce plates from numerical control.

The EPS plate design was treated using a drawing program assisted by a computer and a numerical control application. This way, base blocks were made into a truncated cone shape to avoid possible damage by cutting at the junction of the base (Figure 1c).

In total, the plates have a thickness, similar to those used in construction, of approximately 40 mm. For the realization of the slab mortar, a dosing by weight of 1:3:0.5 (cement:sand:water) has been used, homogenizing the mix with the help of an automatic mixer. Since, when these mortars are made at the worksite, the only conditions taken into account are the flatness and moisture content, the terms of plasticity have not been set for this tests.

The mold used as formwork was wooden, and the dumping and compacting of the pieces were done manually, due to the small size of each of the samples, smoothing the surface with a trowel. Subsequently, pieces in their own mold were introduced into a humid chamber, removing them from the mold 24 h later and keeping the plates in this chamber for another 7 days. The rest of the curing was held outdoors, on laboratory shelves, and all of them were located in the same area, with the idea of avoiding differences due to heat or power in the curing process.

### 2.2. Striking Hammer Design

The fundamental premise followed to design the striking hammer was that all impacts occurred in the most regular and uniform way, fulfilled with the use of a pendulum.

**Figure 2.**
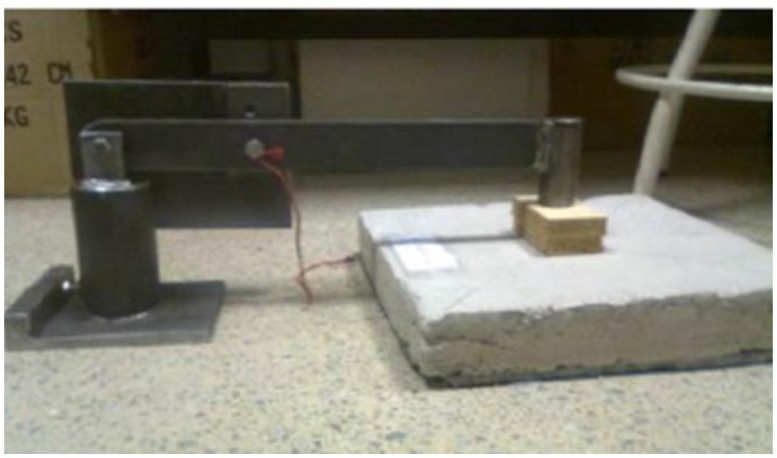
Hammer placed and levelled on wood blocks.

This way, if the hammer shaft is on an axis, the striking will always follow the same path (Figure 2). Then, we set the starting point at the desired height with the help of a bolt that goes through the axis of the hammer. Thus, the falling of the hammer will always be under the same conditions simply by removing the bolt that holds it. Finally, the leveling of the impact point on the tiles will be performed with the help of a few wooden chocks.

The hammer head has a round shape with a 30-mm radius and a sphericity exceeding 500 mm, so that it is in accordance with the standard ISO 9052.

### 2.3. Sensor Test Design

The sensor should be able to determine, once the impact is produced, the power adsorbed by the plate. Knowing the hammer mass and the height of the drop, the potential energy available is known. Once the impact is produced, the plate undergoes a deformation, absorbing part of that power, transmitting the remaining to the adjacent area as impact noise. The plate adsorbs the energy by its own deformation. Thus, the sensor should be able to determine the deformation that occurs on the test plate. To do this, the chosen device was a variable capacitance sensor, capable of measuring the difference that occurs in the capacity of a condenser.

**Figure 3.**
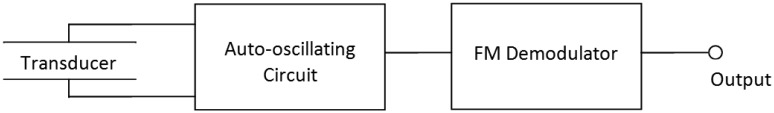
Block diagram of the measurement system.

The sensor is based on the variation of the capacity of two copper plates placed at the bottom of the sample. Between the two copper plates is placed another material, which creates a space between them. The sensor is designed so that the natural oscillation frequency of the self-oscillating circuit is high enough, in our case 3.3 MHz, to detect and measure vibration on the plate from 0 to 20.000 Hz. The distance between copper plates varies in the same way as mechanical oscillations; this variation of the distance transduces the variation of capacity and the change of the output frequency of the self-oscillator circuit. A frequency demodulation is made to obtain the mechanical vibration of the plate. When the strikes occur, the sample deforms, by compressing and pushing the copper plates against each other, thus varying the geometry of the condenser and, therefore, their capacity. In our case, this variation is detected by measuring the oscillation frequency of a self-oscillating circuit. The block diagram of the measurement system can be seen in Figure 3.

## 3. Methodology

Prior to the test, it is necessary to level the plate and adjust the striking hammer, by setting the hammer in a lower position (level at 0° when the hammer head will be placed in contact with the top plate of the sample).

Once the hammer is placed in the fixed position for striking, right in the middle of the sample and forming an angle of 45 degrees to the horizontal, the test begins by dropping the hammer with a single impact over the plate. Upon the impact, the plates of the sensor set under the tile vary their relative position, varying thusly the capacity of the condenser that they form, due to the plate deformation and vibration. The sensor detects this vibration that is transduced as a frequency modulation over the fundamental frequency of the auto-oscillating sensor circuit. By demodulating this signal with the adequate electronic circuit, we obtain the vibration induced in the plate as a result of the hammer impact. Gradually, the deformation will be cushioned, until reaching its starting point.

**Figure 4.**
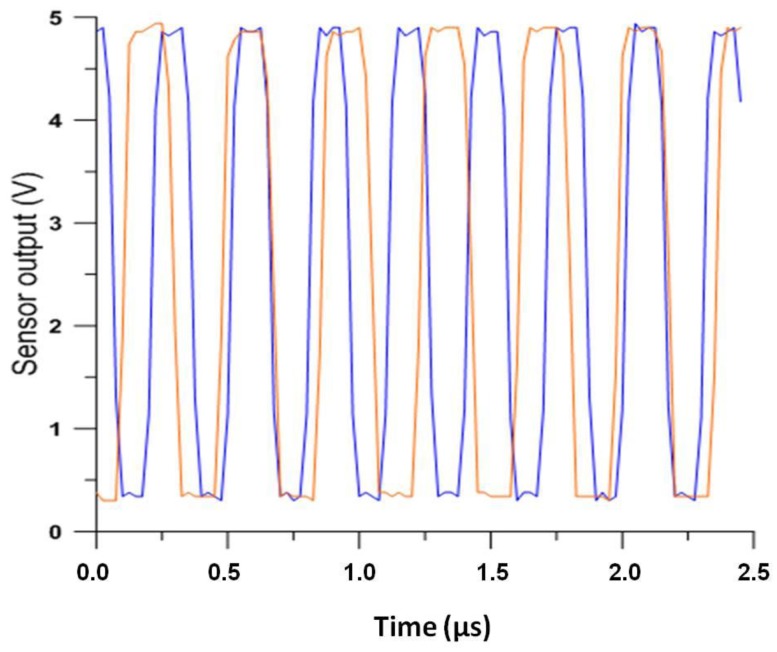
Measurements before (blue) and after (red) the impact.

In Figure 4, the output of the sensor before the hammer impacts over the plate (blue trace) and at the moment immediately subsequent to the impact (red) is shown. It can be see that the frequency output decreases when the sensor detects a deformation due to the impact.

**Figure 5.**
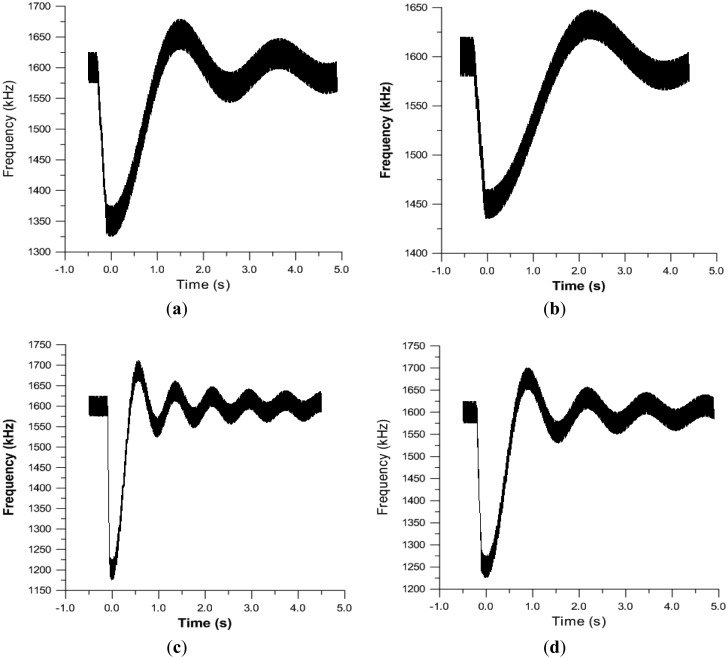
Response curves with four pieces corresponding to different thicknesses of expanded polystyrene (EPS): (**a**) 1 cm; (**b**) 1.5 cm; (**c**) 2 cm; (**d**) 2.5 cm.

The final results obtained, after frequency demodulation of the sensor output, are shown in Figure 5. The greater the deformation of the plates is, the more energy will be absorbed and, therefore, the less energy transmitted as impact noise, as well as the better insulating it will be. The biggest jump in frequency will be the deformation indicator and, therefore, the absorbed power.

## 4. Results and Discussion

The measurements, as already mentioned, were carried out, and each measurement covered 5 s, which is about eight million periods of the signal at the frequency sensor base. This ensures, on the one hand, enough time for acquisition to pick up the response of the material and, on the other hand, guarantees that the number of samples per period is enough to determine with precision the variations in frequency.

From these measurements, the frequency response is obtained as a function of time, which is the material response to the received strike. Below are four response curves (Figure 4) with different thicknesses of EPS (1, 1.5, 2, 2.5 cm), corresponding to four plates, in which, for all of them, the zero has been set at the time of the impact.

These graphs show that the methodology tested is able to obtain answers from the different analyzed samples, and the answers obtained show different temporal behaviors (and therefore, different spectral responses) that could be correlated with their acoustic behavior.

In addition, as can be seen in Figure 4, the obtained frequencies are affected by considerable noise. This is due to the method of obtaining them. This can be solved by improving the electronic demodulator, which would enable capturing the signals with less noise and better resolution.

## 5. Conclusions

From the data obtained and the significant graphics represented, the moment at which the impact has been produced on each of the plates can be clearly seen. Furthermore, after this impact, there is a temporary response, and therefore, the spectrum thereof could be obtained.

On the other hand, the response of the plate is clearly distinguishable in the test, as a constant frequency behavior is observed from the beginning of the same until the moment of impact, in which a very pronounced drop and rise takes place, after which, there is a gradually recovery, until reaching a frequency equal to the initial one.

All of this leads to considering the possible applications of this sensor, as a method to be used for obtaining the parameters for sound insulation materials for floors, which can range from rehearsals aimed at acoustic normalization of prefabricated tiles, to integration of in more complex structures, such as an alarm system.
